# Activated hepatic stellate cells promote liver cancer by induction of myeloid-derived suppressor cells through cyclooxygenase-2

**DOI:** 10.18632/oncotarget.6839

**Published:** 2016-01-07

**Authors:** Yaping Xu, Wenxiu Zhao, Jianfeng Xu, Jie Li, Zaifa Hong, Zhenyu Yin, Xiaomin Wang

**Affiliations:** ^1^ Department of Hepatobiliary Surgery, Zhongshan Hospital, Xiamen University, Fujian Provincial Key Laboratory of Chronic Liver Disease and Hepatocellular Carcinoma (Xiamen University Affiliated Zhongshan Hospital), Xiamen, Fujian, China; ^2^ Department of Basic Medicine, Xiamen Medicine College, Fujian, China

**Keywords:** myeloid-derived suppressor cells, hepatic stellate cell, cyclooxygenase 2, hepatocellular carcinoma, prostaglandin 2

## Abstract

Hepatic stellate cells (HSCs) are critical mediators of immunosuppression and the pathogenesis of hepatocellular carcinoma (HCC). Our previous work indicates that HSCs promote HCC progression by enhancing immunosuppressive cell populations including myeloid-derived suppressor cells (MDSCs) and regulatory T cells (Tregs). MDSCs are induced by inflammatory cytokines (e.g., prostaglandins) and are important in immune suppression. However, how HSCs mediate expansion of MDSCs is uncertain. Thus, we studied activated HSCs that could induce MDSCs from bone marrow cells and noted that HSC-induced MDSCs up-regulated immunosuppressive activity *via* iNOS, Arg-1, and IL-4Rα. After treating cells with a COX-2 inhibitor or an EP4 antagonist, we established that HSC-induced MDSC accumulation was mediated by the COX2-PGE_2_-EP4 signaling. Furthermore, *in vivo* animal studies confirmed that inhibition of HSC-derived PGE_2_ could inhibit HSC-induced MDSC accumulation and HCC growth. Thus, our data show that HSCs are required for MDSC accumulation mediated by the COX2-PGE_2_-EP4 pathway, and these data are the first to link HSC and MDSC subsets in HCC immune microenvironment and provide a rationale for targeting PGE_2_ signaling for HCC therapy.

## INTRODUCTION

Hepatocellular carcinoma (HCC) is one of the most malignant tumors and the third leading cause of cancer-related deaths around the world [1, 2]. Hepatocarcinogenesis is a complex and multi-factorial process and at this time, some molecular pathways have been identified that contribute to the development, progression, angiogenesis, and metastasis of HCC. The tumor microenvironment is of interest in cancer research and is now recognized as a critical contributor to cancer progression. Infiltrating stromal and immune cells in the tumor microenvironment contribute to cancer biology as well [3].

Hepatic stellate cells (HSCs) are important stromal cells that are activated during liver injury, inflammation, infection, trauma, or by other pathogens [4, 5]. HSCs have immunomodulatory activity and prolong islet allografts survival [6, 7]. We reported that activated HSCs can induce immune-suppressing cells, specifically regulatory T cells (Tregs) and myeloid-derived suppressor cells (MDSCs) in tissues of tumor-bearing mice [8-10]. MDSCs are heterogeneous immature myeloid cells comprised of myeloid progenitors and precursors of macrophages, granulocytes, and dendritic cells (DCs) [11, 12]. Murine MDSCs co-express CD11b and Gr-1 [11] and their heterogeneity is also based on expression of Gr-1: granulocytic-MDSCs (G-MDSCs, CD11b^+^Ly6G^+^Ly6C^int/low^), and monocytic-MDSCs (Mo-MDSCs, CD11b^+^ Ly6G^−^Ly6C^high^) [13, 14]. Höchst's group reported that activated HSCs can recruit and transform peripheral blood monocytes into *de novo* MDSCs [15] but how HSCs induce MDSC expansion and activation is unclear.

Recently, Qian and colleagues reported that HSCs induced MDSCs via soluble factors secreted by HSCs and expanded these data by revealing that complement component 3 (C3) is critical for inducing MDSC expansion and protecting islet allografts [16]. However, HSCs deficient in C3 did not completely lose their capacity to induce MDSCs, implying the involvement of other factors that may synergize with C3 to promote MDSC induction. Vascular endothelial growth factor (VEGF), granulocyte macrophage colony-stimulating factor (GM-CSF), and granulocyte colony-stimulating factor (G-CSF) promote MDSC activity in cancer [11, 17] but Qian et al has proved that these factors do not involved in induction of MDSC [7], so additional factors are required for induction of MDSCs by HSCs.

Prostaglandin E2 (PGE_2_) is a pro-inflammatory mediator produced by cancer, stromal, and infiltrating myeloid cells and acts on G-protein-coupled receptors (GPCRs) including EP1-EP4 [18]. Cyclooxygenase (COX)-2 is chiefly believed to be key to influencing the rate of PGE_2_ production during immune response [19]. A positive feedback loop between PGE_2_ and COX-2 determines the redirection of the development of CD1a^+^ DCs to CD1a^−^CD14^+^CD80^−^CD83^−^ monocytic MDSCs [20]. Furthermore, Kalinski's group reported that addition of PGE_2_ to GM-CSF/IL-4-supplemented monocytic precursor cultures generated numerous MDSCs [21]. Silencing *COX-2* in 4T1 tumor cells reduced CD11b^+^Gr-1^+^ MDSC accumulation in mouse spleens [11]. Moreover, PGE_2_ can be produced by HSCs [22-24], which suggested the hypothesis that HSCs induce expansion of MDSCs via secreted PGE_2_. For this reason, bone marrow (BM) cells were cultured with HSC-conditioned medium (HSC-CM) plus SC-236, a COX2 inhibitor. Then, the effect of SC-236-treated HSCs on MDSC expansion and tumor growth was assessed.

## RESULTS

### Incubation of BM cells with conditioned media from activated HSCs induced MDSCs

**Figure 1:**
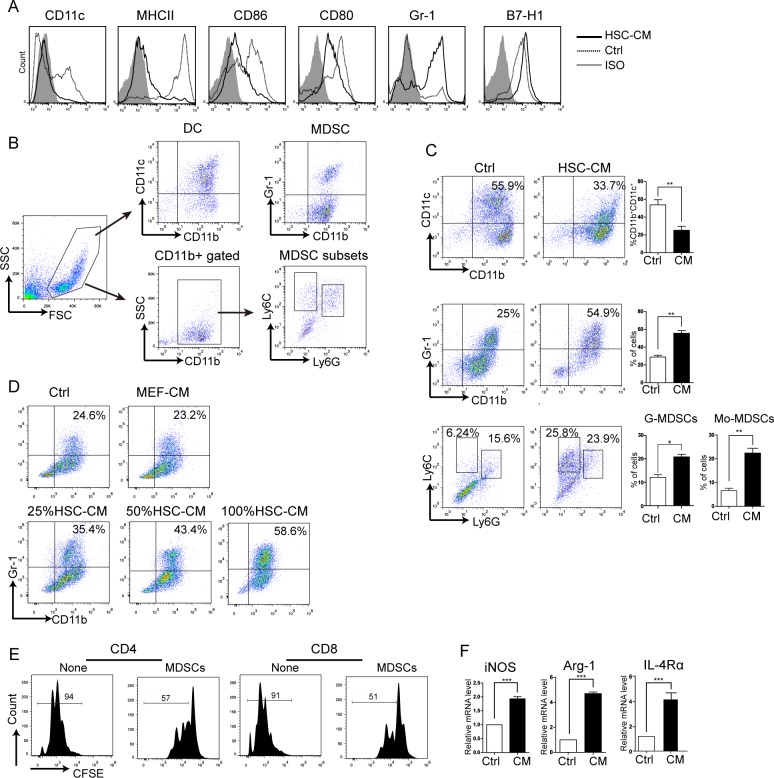
Effects of HSC-CM on BM-derived DC differentiation *in vitro* **A.** Cell surface marker expression in myeloid cells after HSC-CM treatment measured by flow cytometry. Filled areas are isotype controls; dotted lines are RPMI 1640 medium controls; full lines are HSC-CM. **B.** Gating strategy of DCs, total MDSCs and subsets. **C.** The effect of HSC-CM on DCs, MDSCs and MDSC subgroups. Number is percent of the cell population represented by immature DCs (top panel), MDSCs (middle panel) or Mo-MDSCs, G-MDSCs (bottom panel). Percent G-MDSCs was calculated as follows: corrected G-MDSC percent = 100% × CD11b^+^ percent × Ly6G^+^Ly6C^low^ percent. Corrected Mo-MDSC percent = 100% × CD11b^+^ percent × Ly6G^−^Ly6C^high^ percent. **D.** HSC-CM induced MDSCs in a concentration-dependent manner. MEF-CM was a control CM. **E.** Gr-1^+^ cells inhibited T-cell proliferation. **F.** *iNOS*, *Arg-1*, and *IL-4Rα* expression in Gr-1^+^ cells according to RT-PCR. Data represent 3-5 independent experiments and are expressed as means ± SD; *P < 0.05, **P < 0.01, ***P < 0.001.

First, BALB/c BM cells were cultured with HSC-CM and cell surface marker expression by various myeloid cell types after HSC-CM treatment was measured. Figure 1A shows BM co-cultured with HSC-CM decreased CD11c, MHC II, CD86 and CD80 expression, suggesting less BM cell differentiation into macrophages and immature DCs. Meanwhile, Gr-1 increased significantly and a slight increase in B7-H1.

Next, the expression of DCs, MDSCs, and MDSC subsets were detected after BM cells were cultured with HSC-CM. The gating strategy of these cells is shown in Figure 1B. As shown in the Figure 1C, the number of CD11b^+^CD11C^+^ DCs decreased by approximately half (decreased from 55.9 ± 2.1% to 33.7 ± 1.9%, *P* < 0.01. Figure 1C, upper panel). CD11b/Gr-1 co-staining confirmed the presence of MDSCs, which doubled (25 ± 2.9% in control *vs.* 54.9 ± 2.4% in HSC-CM, Figure 1C, middle panel). MDSCs can be divided phenotypically into granulocytic (MDSCs, CD11b^+^/Ly6G^+^/Ly6C^int/low^) and monocytic (Mo-MDSCs, CD11b+/Ly6G^−^/Ly6C^high^) subgroups, which have been shown to be immunosuppressive via different pathways. Figure 1C (bottom panel), depicts G-MDSCs and Mo-MDSCs induction in HSC-CM culture and that upregulation of Mo-MDSC was most prominent and these findings agree with those of Qian's group [16]. To verify the specific HSC-CM effect on MDSC expansion, we used CM from MEF cells as controls and noted that MEF-CM had no effect on MDSC expansion, and the influence of HSC-CM on BM cells was concentration-dependent (Figure 1D).

To study immunosuppression of MDSCs, Gr-1^+^ cells were isolated using MACS, and more than 90% of the Gr-1^+^ cells were CD11b^+^Gr-1^+^. MDSCs were cultured with T cells (1:1). As shown in Figure 1E, MDSCs inhibited T-cell proliferation. It has been reported that elevated expression of Arg-1, iNOS, and IL-4Rα accounts for suppression of T-cell function by MDSCs [11, 25]. For this reason, the mRNA expression of each protein was detected, and a 2-fold increase in *iNOS* mRNA, a 4.5-fold increase in *Arg-1* mRNA, and a 4-fold increase in *IL-4Rα* expression were detected (Figure 1F). In this way, HSC-CM inhibited DC development and promoted MDSC accumulation *in vitro*.

### Induction of MDSCs from BM cells by HSC-conditioned media is mediated by PGE2 production

**Figure 2:**
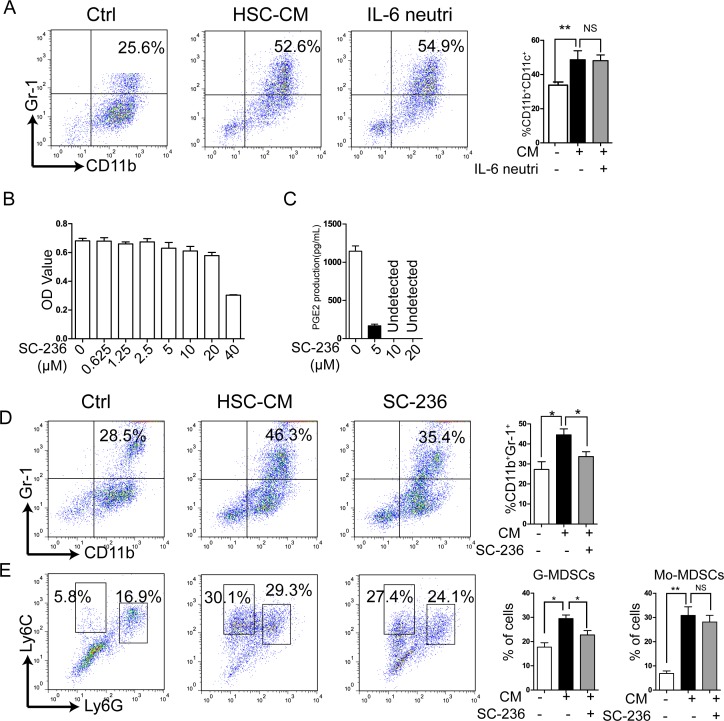
Inhibition of PGE_2_ production by HSCs reversed the effect of HSC-CM on MDSC induction *in vitro* **A.** Effect of IL-6 neutralization on MDSC expression. Number represents percent of MDSCs in entire cell population (right panel). **B.** Cell counts according to CCK8 assay. **C.** PGE_2_ secretion measured by ELISA. **D.**-**E.** MDSC, G-MDSC, and Mo-MDSC populations assessed with flow cytometry. Number represents percent of entire cell population (right panels), calculated as described in Figure 1. Number is percent of the cell population represented by Mo-MDSCs and G-MDSCs. Data are means of 3-5 independent experiments ± SD; *P < 0.05, **P < 0.01, ***P < 0.001.

Factors such as cytokines (IL-6) [26, 27], stem-cell factor (SCF) [28], growth factors VEGF, TGF-β, GM-CSF, G-CSF, and macrophage colony-stimulating factor (M-CSF), COX-2, and PGE_2_ [21, 29, 30] have been reported to induce MDSC expansion. IL-6 is abundantly produced by activated HSCs according to previous work from our laboratory and that of others [9], so we speculated that HSCs may induce MDSC accumulation via IL-6. To test this hypothesis, IL-6 neutralizing antibody was added to HSC-CM cultures of BM cells and MDSCs did not decrease compared to the HSC-CM group (Figure 2A), so IL-6 does not apparently mediate the effect of HSC-CM on MDSC induction.

PGE_2_ influences MDSC expansion and is secreted by activated HSCs [23, 24], so to assess whether HSC-CM induced MDSC expansion was correlated with PGE_2_ production, HSCs were pretreated with SC-236, a COX-2 inhibitor. Figure 2B depicts no significant cytotoxicity of SC-236 against HSCs (5-20 μM), but PGE2 production was inhibited by SC-236 (Figure 2C).

BM cells were cultured with the HSC-CM or conditioned media from 20 μM SC-236-pretreated HSCs (SC-236-CM) for 3 days. Flow cytometry indicated a significant increase of MDSCs in the HSC-CM group compared to controls (Figure 2E). SC-236-CM significantly reduced MDSCs induced by HSC-CM (Figure 2D), suggesting the importance of PGE_2_ in inducing MDSCs by HSC-CM.

We next investigated the effect of SC-236 on MDSC subgroups. HSC-CM upregulated expression of both CD11b^+^Ly6G^+^Ly6C^int/low^ G-MDSCs and CD11b^+^Ly6G^−^Ly6C^high^ Mo-MDSCs, especially Mo-MDSCs (Figure 2E). SC-236-CM abolished G-MDSCs promotion by HSCs, and they decreased in the HSC-CM and SC-236-CM groups. However, the effect of HSCs on Mo-MDSCs induction was not altered significantly by SC-236 treatment, suggesting that induction of Mo-MDSCs was independent of PGE_2_ production in HSCs.

### EP4 antagonists block MDSC accumulation

**Figure 3:**
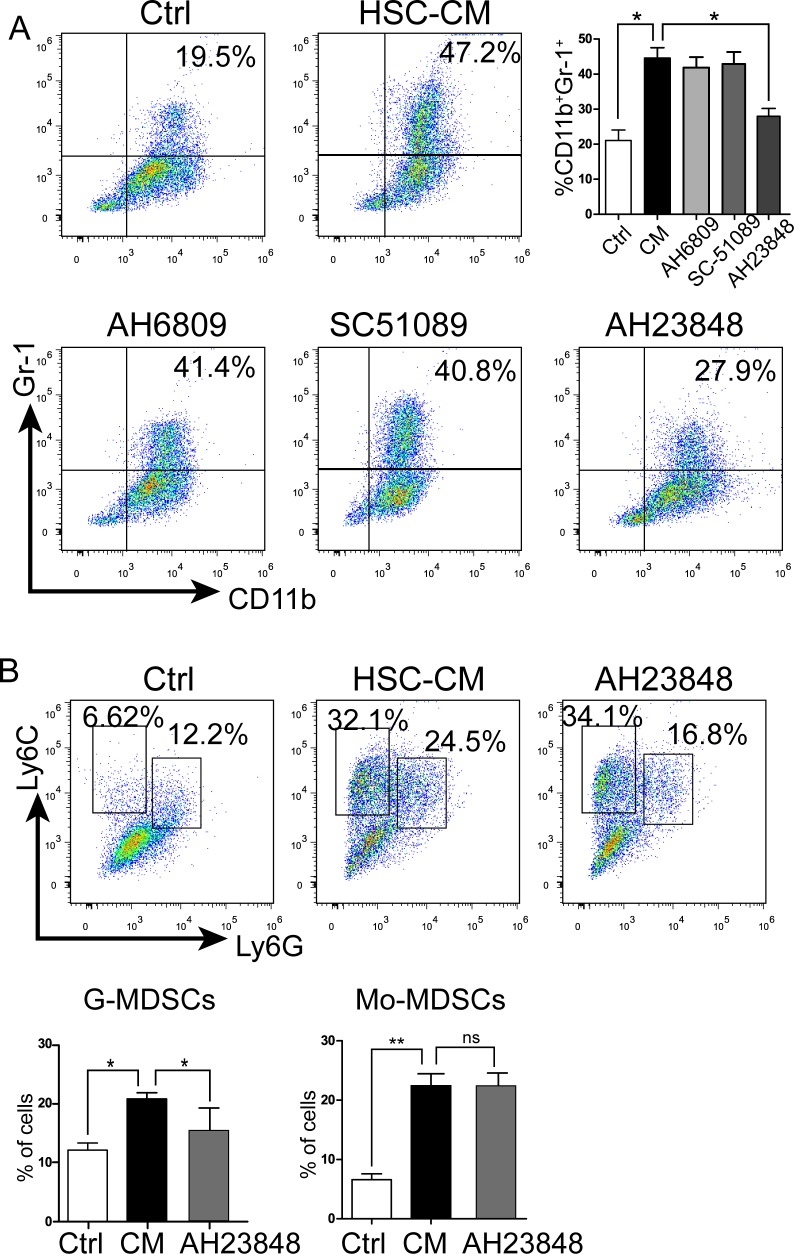
HSCs promoted MDSC accumulation via the EP4 receptor **A.** Effect of EP antagonists on MDSC expression. Number is percent of the cell population represented by MDSCs (EP1 antagonist SC-51089; EP1 and EP2 antagonist AH6809; EP4 antagonist AH23848). **B.** Expression of G-MDSCs and Mo-MDSCs in BM cells cultured with/without EP4 receptor antagonist AH23848; number is percent of the cell population represented by Mo-MDSCs and G-MDSCs as calculated in Figure 1. Data are means of 3-5 independent experiments ± SD; *P < 0.05, **P < 0.01, ***P < 0.001.

MDSCs from 4T1 tumor-bearing mice are known to express four E-prostanoid receptors (EP) for PGE_2_ [30]. To validate that HSCs induces MDSC accumulation via the PGE_2_/EP signaling way, BM cells were cultured with IL-4 and GM-CSF with/without CM, or with/without EP antagonists. Figure 3A shows that HSC-CM upregulated expression of MDSCs. When BM cells were cultured with HSC-CM plus EP antagonists, only the EP4 antagonist inhibited induction of MDSCs by HSC-CM. Data show that accumulation of MDSCs induced by HSCs was mediated through the PGE_2_/EP4 signaling.

Because the stimulatory effect of HSCs on Mo-MDSCs only slightly decreased with SC-236 treatment. Next, experiments were conducted to determine whether the G-MDSC and Mo-MDSC subsets induced by HSCs were regulated by the PGE_2_/EP4 signaling. As shown in Figure 3B, AH23848 (EP4 antagonist) inhibited the G-MDSC but not Mo-MDSC expansion by HSC-CM. Taken together, these *in vitro* data indicated that HSC-derived PGE_2_ induced the expansion and differentiation of MDSCs, especially the G-MDSC subset, through the EP4 receptor.

### Inhibition of COX-2 activity in HSCs impairs MDSC induction *in vivo*

**Figure 4:**
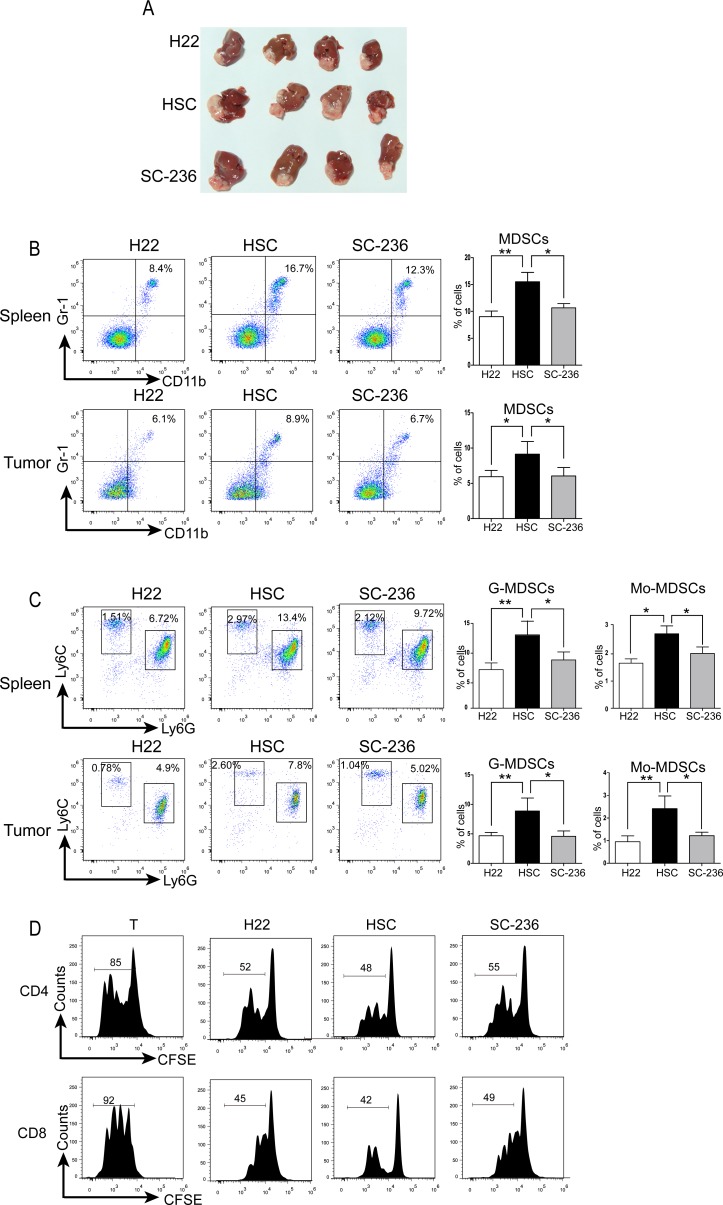
HSCs promoted HCC growth by inducing MDSCs *via* PGE_2_ signaling **A.** Representative tumors. **B.** MDSC accumulation in splenocytes and tumors were counted; number is percent of the cell population represented by MDSCs (right panels). **C.** G-MDSCs and Mo-MDSCs were measured with flow cytometry. Number is percent of the cell population represented by Mo-MDSC and G-MDSC, calculated as described in Figure 1. **D.** CD4+ and CD8+ T cells measured by CFSE dilution assay and flow cytometry. Data are means ± SD; *P < 0.05, **P < 0.01, ***P < 0.001.

To understand how HSCs-produced PGE_2_ affects accumulation of MDSC cells *in vivo*, a murine orthotopic HCC model was established. The liver capsule was injected with H22 cells alone (1 × 10^6^ cells/mouse, control group), or H22 plus HSCs (2 × 10^5^ cells/mouse, HSC co-transplanted group), or H22 and SC-236-pretreated HSCs (2 × 10^5^ cells/mouse, SC-236 pretreated group) (n = 8/group). Figure 4A shows that tumors from the HSC co-transplanted group were significantly larger than those of controls, a finding that agreed with previous observations [10]. In addition, tumors were smaller in the SC-236 pretreated group compared to the HSC co-transplanted group, suggesting that HSC pretreated with SC-236 lost tumor-promoting activity.

Because the primary tumor burden by itself does not dictate the level of MDSCs [30], we then further investigated the accumulation of MDSCs in the spleen and inside the tumor among the 3 groups *via* flow cytometric analysis (Figure 4B). Significant differences in the percent of MDSCs in the spleen between control group and HSC co-transplanted group with or without SC-236 pretreatment were observed (8.4% *vs.* 16.7% for control *vs.* HSC co-transplanted group, *P* < 0.01; 16.7% *vs.* 12.3% for HSC co-transplanted group *vs.* SC-236 pretreated group, *P* < 0.05). Further analysis of MDSCs population in the tumors revealed a similar pattern. As shown in Figure 4B (bottom panel), there was a significant increase in the percent of MDSCs in the tumor in the HSC co-transplanted group relative to the H22 alone group (8.9% *vs.* 6.1%; *P* < 0.05). HSCs pretreated with SC-236 resulted in a 25% decrease in the MDSC population as compared to that observed in the HSC group (*P* < 0.05).

Next, we sought to evaluate the subsets of MDSCs in tumors co-transplanted with HSCs or SC-236-pretreated HSCs. As Figure 4C shows, co-transplanted HSCs significantly increased both the percent of G-MDSCs and Mo-MDSCs in spleen and tumor, whereas HSCs pretreated with SC-236 presented a reduction in G-MDSCs and Mo-MDSCs expression both in the spleen and tumors, respectively. To evaluate MDSC immunosuppressive activity, MDSCs were isolated from the control group, HSC co-transplanted group, and SC-236 pretreated group. As shown in Figure 4D, MDSC immunosuppressive activity showed no obvious difference among three groups. The data from the subcutaneous model (Supplementary Figure 2) were consistent with the orthotopic HCC model. To confirm whether the increase in MDSCs in the HSC group was due to the higher tumor burden of these mice, the population of MDSCs was assessed in the spleens of mice at 1 week, 2 weeks, and 3 weeks after tumor injection. The data show that the MDSCs in the HSCs were higher than those in the control group and SC-236 group since at the first week when tumors did not different in size among groups (Supplementary Figure 3). These findings suggest that COX-2 antagonist SC-236-pretreated HSCs could reduce the tumor size and the population of MDSCs in tumor-bearing mice.

## DISCUSSION

Researchers across the world have made great strides in understanding the role of the tumor environment in tumor progression, but cross-talk between stromal and immune cells in cancer progression and metastasis is entirely unexplored. Our previous work indicates that activated HSCs can create an immunosuppressive microenvironment in an orthotopic liver tumor mouse model by inducing expansion of Tregs and MDSCs [10], but how this occurs is unclear. Qian's group reported that MDSCs could be induced by HSC derived C3 to protect islet allografts, but HSCs deficient in C3 did not completely lose their capacity to induce MDSCs [16]. Here, we investigated other factors involved in induction of MDSCs and measured their effects on specific subsets of MDSCs and noted that HSCs promoted MDSC accumulation, particularly Mo-MDSCs. Furthermore, data showed that HSCs promoted G-MDSC expansion via COX2-PGE_2_-EP4 signaling, and inhibiting HSC-derived PEG_2_ with a COX-2 inhibitor reduced tumor grow and MDSCs accumulation, which indicated that the PGE_2_/EP4 pathway was involved in induction of MDSCs by HSCs.

MDSCs are critical mediators of tumor-induced immune dysfunction and cancer progression [32]. Previous work indicates that MDSC expansion is regulated by factors such as COX2, PGE2, SCF, M-CSF, IL-6, GM-CSF, and VEGF [11, 28, 30, 33], most of which are produced by activated HSCs. IL-6, VEGF, GM-CSF, and G-CSF have been implicated in MDSC development [6, 34-37]. Reports suggest that induction of MDSCs by HSCs is unlikely to be mediated by VEGF, GM-CSF, or G-CSF [7, 16]. IL-6 is sufficient to drive MDSC expansion, compared to other factors produced by various tumors and it causes a rapid generation of MDSCs from mouse and human BM [38-40]. In BM cell culture, however, IL-6 neutralizing antibody had little or no effect on generation of CD11b^+^Gr-1^+^ MDSCs (Figure 2A). PGE_2_ is known to be a regulator of MDSC accumulation, MDSC-mediated T cell inhibition, and expression of Arg1 and iNOS [41-43]. Evidence is mounting that activation of COX-2 signaling results in an alteration of HSCs [44, 45]. In addition, COX-2 and COX-2-dependent PGE_2_ expression was persistently upregulated in fully activated HSCs and participated in the chemokines regulation, VEGF production, and HSC proliferation and migration [46-48]. PGE_2_ also induces MDSC expansion [21, 49-54], so activated HSCs that produce abundant PGE_2_ were evaluated next.

The expression and expansion of MDSCs were reduced after treatment of HSCs with COX-2 inhibitor SC-236, which indicated that MDSC induction by HSCs was mediated by the COX-2/PGE_2_ pathway. IFN-γ has been reported to be critical for HSC-mediated MDSC generation [7] and HSCs deficiency in IFN-γ R1 largely lose the ability to induce MDSCs. PGE_2_ signaling is thought to mobilize the cAMP-PKA pathway via EP2/EP4 receptors and induce CREB- and its co-activator CRTC2-mediated transcription of IL-12Rβ2 and IFN-γR1 [55]. Therefore, inhibiting PGE2 signaling may affect transcription of IFN-γ R1, thereby decreasing MDSCs.

We assessed the effect of COX2 inhibitor SC-236 on MDSC subset generation and noted that only G-MDSC expansion decreased with SC-236 CM but not Mo-MDSCs. PGE_2_ exerts its functions by interacting with PGE_2_ receptors, of which there are four subtypes (EP1-4) [56]. PGE_2_ is reported to induce accumulation of Gr-1^+^CD11b^+^ MDSCs from BM cells via EP1, EP2, and/or EP4 receptors [30]. In our study, only an EP4 receptor antagonist inhibited MDSC expansion and G-MDSC expansion appeared to be the only cell type affected, although both G-MDSCs and Mo-MDSCs expressed the EP4 receptor (Supplementary Figure 1). Thus, expansion of G-MDSCs induced by HSCs was through the COX2-PGE_2_-EP4 signaling pathway and different factors might be involved in Mo-MDSC expansion.

Qian's group reported that BM cells cultured with HSCs deficient in C3 expressed less Ly6C compared to wild type HSCs, suggesting that C3 may be crucial for inducing Mo-MDSC (CD11b^+^ Ly6G-Ly6C^high^ cell) expression [16]. Also shRNA-mediated reductions in C3 expression in HSCs impaired Mo-MDSC expression (data not shown). Possibly, C3 has greater priority over PGE_2_ for inhibiting Mo-MDSC expression. SC-236 had no obvious effect on immunosuppressive activity of MDSCs isolated from tumors, perhaps because of the abundant inflammatory factors secreted by tumor cells that stimulate immunosuppressive activity of MDSCs. TGF-β1-induced miR-494 expression in MDSCs is essential for accumulation and function of tumor-expanded MDSCs [57].

In our animal model, reduction of HSC-derived PGE_2_ with the COX2 inhibitor reduced Gr-1^+^ CD11b^+^ cells accumulation and decreased growth of implanted tumors. Interestingly, HSCs pretreated with COX2 inhibitor could better inhibit Mo-MDSC accumulation *in vivo*, although the COX2 inhibitor and the EP receptor antagonist had less effect on Mo-MDSC accumulation in co-culture. This may be explained by differences in the tumor environment. Fewer Mo-MDSCs *in vivo* may result from differentiation of Mo-MDSCs into other types of immune cells. Ly6C expression in Mo-MDSC sorted from the spleen of EL-4 TB mice was reported to be down-regulated expression by ∼30% after 3 days of culture with GM-CSF [58].

Overproduction of COX2 and PGE_2_ in the tumor microenvironment may also explain the local accumulation of MDSCs observed in different cancers [32, 59]. HSC co-transplantation induced more PGE_2_ expression than control group, while co-transplantation with COX-2 inhibitor-pretreated HSCs produced a PGE_2_-reduced microenvironment than in the HSC co-transplantation group, which reduced Mo-MDSC accumulation (Supplemental Figure 4), However, HSC-secreted PGE_2_ influenced MDSC accumulation but not their immunosuppressive activity. Different chemokine/chemokine receptor signal axes play different roles in mediating the mobilization of MDSC subsets. The CXCR4-CXCL12 axis is necessary for mediating the attraction of monocytic MDSCs into the tumor environment and PGE_2_ is important to regulation of CXCL12 production in cancer-associated fibroblasts and the cancer environment, and to enhancement of CXCR4 expression in Mo-MDSCs [29]. CCL2 has been reported to foster PMN-MDSC accumulation in tumors [60]. The expression of *Cxcl12* and *Ccl2* was also measured in the tumor microenvironment, and data show the expression of *Cxcl12* and *Ccl2* to be increased in the HSC co-transplantation group. Relative to the HSC co-transplantation group, *Cxcl12* and *Ccl2* mRNA expression was down-regulated in the SC-236 group.

**Figure 5:**
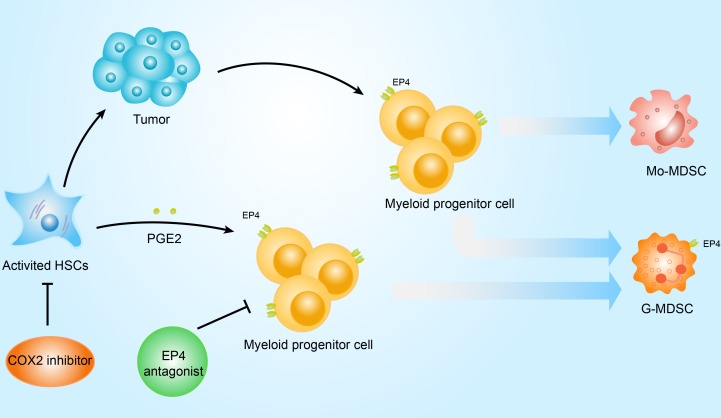
Scheme of MDSC induction signaling pathway by HSCs *In vitro*, activated HSC-derived PGE_2_ inhibits myeloid progenitor cell maturity and induction of G-MDSC accumulation via the EP4 receptor, which can be reversed with a selective COX2 inhibitor or EP4 antagonist. Inhibition of activated HSC-derived PGE2 reduces HCC growth and HSC-induced MDSC accumulation.

Our data indicate a relationship between activated HSCs and MDSC subsets in the HCC tumor microenvironment. HSCs promote G-MDSC accumulation via COX2-PGE2-EP4 signaling and COX2 inhibition blocks HSC-derived PGE_2_ and HSC-mediated induction of MDSCs, decreasing HSC tumor-promoting ability (Figure 5). We offer a novel mechanistic explanation for the link between HSCs and HCC progression, targeting PGE_2_ as an innovative strategy for future cancer immunotherapy development.

## MATERIALS AND METHODS

### Animals and cell lines

All experimental protocols were reviewed and approved by our Institutional Review Board and animal experimental protocols were performed in compliance with the Guidelines for the Institutional Animal Care and Use Committee of Xiamen University.

Adult male BALB/c mice (H-2d, haplotype, 8-12 weeks-of-age) mice were purchased from the National Rodent Laboratory Animal Resources, Shanghai, China. The mouse H22 hepatoma cell line was purchased from Shanghai Cell Bank, Chinese Academy of Sciences, and maintained in RPMI 1640 medium (HyClone, Logan, UT), supplemented with 10% fetal bovine serum (FBS), 100 U/mL penicillin, and 100 μg/mL streptomycin, as previously described [9].

### HSCs isolation and culture

HSCs were isolated from BALB/c mouse livers as described previously [9]. Livers were perfused with phosphate buffered saline (PBS) and type IV collagenase (Sigma, city, state), and soaked in collagenase for further digestion. Isolated HSCs were cultured in RPMI 1640 medium (HyClone, Logan, UT, U.S.) supplemented with 10% heat-inactivated FBS (Gibco, BRL Co. Ltd.), 100 U/mL penicillin, and 100 mg/mL streptomycin in 5% CO_2_/95% air at 37°C. Cells at passage 4-10 were used for experiments. Cell activation was measured via a-SMA staining [9, 31].

### Culture of DCs

DCs were prepared according to published methods [31]. After lysing RBCs with lysis buffer (Beyotime, Jiangsu, China), 1 × 10^6^ BM cells/well from tibias and femurs of BALB/c mice were cultured in RPMI 1640 medium containing 10% FBS and mouse recombinant GM-CSF (10 ng/mL, R&D, Minneapolis, MN, U.S.; IL-4 10 ng/mL, PeproTech, Rocky Hill, NJ, U.S.) for 6 days. To measure the effect of HSCs on BM, (HSC-CM) or SC-236, CM was used to replace RPMI 1640 medium at DC culture initiation. Floating cells were harvested, washed, and resuspended in RPMI 1640 medium.

### Isolation of MDSCs

Tumors or spleen were cut into small pieces and dissociated with a MACS dissociator (Miltenyi Biotec, Bergisch Gladbach, Germany) according to the manufacturer's instructions. After dissociation, dead cells were excluded by Dead Cell Removal Kit (Miltenyi Biotec, Bergisch Gladbach, Germany). Then, the remaining cells were used to isolate MDSCs by bition-Gr-1 antibody (BD, San Diego, CA, U.S.) and Streptavidin MicroBeads (Miltenyi Biotec).

### Mixed leukocyte reaction

A mixed leukocyte reaction (MLR) culture was performed in 24-well plates (Corning, Corning, NY). Nylon wool-eluted splenic T cells (1 × 10^6^/well) from BALB/c mice were used as responders. T-cell proliferation was elicited with anti-CD3 mAb (1 μg/mL) and CD28 (1 μg/mL). Cultures were maintained in RPMI 1640 complete medium for 3 days under 5% CO_2_. T-cell proliferation was measured with a CFSE dilution assay and flow cytometry.

### Tumor inoculation

Mice were injected intra-hepatically with 0.1 mL cell suspension containing either 1 × 10^6^ H22 cells or a mixture of 1 × 10^6^ H22 cells and 2 × 10^5^ activated HSCs (N = 8/group). To understand the role of HSC-derived PGE_2_, HSCs were pretreated with the COX2 antagonist SC-236 (20 μM) 3 days before inoculation. HSCs treated with DMSO were controls. At the end of the experiment, mice were euthanized, and spleens and tumors were collected and stored for subsequent analysis.

### CCK8 and PGE_2_ assay

Cells were treated with different concentration of SC-236 for 24 h, and 10 μl CCK8 reagent was added. After 4 h, absorbance was read (λ 450 nm). A PGE_2_ ELISA kit was obtained from R&D Systems and assays were conducted according to kit instructions.

### Quantitative real-time PCR assay

Total RNA was extracted with TRIzol Reagent (Invitrogen, city, state). Complementary DNA (cDNA) was synthesized with SuperScript II reverse transcriptase (Invitrogen). Quantitative PCR (qPCR) primers were as follows: *Arginase-1*: Forward: 5′-CACGGCAGTGGCTTTAACCT-3′, Reverse: 5′-TGGCGCATTCACAGTCACTT-3′; *iNOS*: Forward: 5′-GGAATCTTGGAGCGAGTTGT-3′, Reverse: 5′- CCTCTTGTCTTTGACCCAGTAG-3′. *IL-4Rα*: Forward: 5′-CCTACACTACAGGCTGATGTTC-3′, Reverse: 5′-TGGACCGGCCTATTCATTTC-3′. mRNA was measured with a 7500 Fast PCR system (Applied Biosystems, Foster City, CA, U.S.) in duplicate and were normalized to GAPDH mRNA.

### Flow cytometry

Splenocytes were harvested and disaggregated in 10 mL of RPMI 1640 complete medium. RBCs were lysed with RBC Lysis Buffer (Beyotime, Nanjing, China). Tumors were cut into small pieces and dissociated with a MACS dissociator (Miltenyi Biotec, Bergisch Gladbach, Germany) according to the manufacturer's instructions. After dissociation, dead cells were exclude with DAPI (0.2 μg/mL), and leukocytes were gated on FSC and SSC for analysis. Monoclonal antibodies against CD11b, CD11C, Ly6C, Ly6G, and Gr-1 were purchased from BD PharMingen (San Diego, CA, U.S.), and antibodies against CD40, CD86, CD80, MHCI, MHCII, and B7-H1 were purchased from eBioscience (San Diego, CA, U.S.). Fluorescently-labeled cells were analyzed with flow cytometry (Beckman Gallios). Analysis and graphical output were performed using with FlowJo software and appropriate isotype control antibodies were used.

### Statistical analysis

Data were analyzed using SPSS software (version 13.0) and are expressed as means ± SD. Statistical analyses were performed with a Student's *t* test (*P* < 0.05).

## SUPPLEMENTARY MATERIAL FIGURES


