# Supplementary material for: Activated hepatic stellate cells promote liver cancer by induction of myeloid-derived suppressor cells through cyclooxygenase-2
Source: Oncotarget. 2016 Jan 7;7(8):8866–78. doi: 10.18632/oncotarget.6839 (PMC4891010; doi:10.18632/oncotarget.6839)
Supplement: Supplementary file 1 [file oncotarget-07-8866-s001.pdf]

Activated hepatic stellate cells promote liver cancer by induction of myeloid-derived suppressor cells through cyclooxygenase-2

Supplementary Material

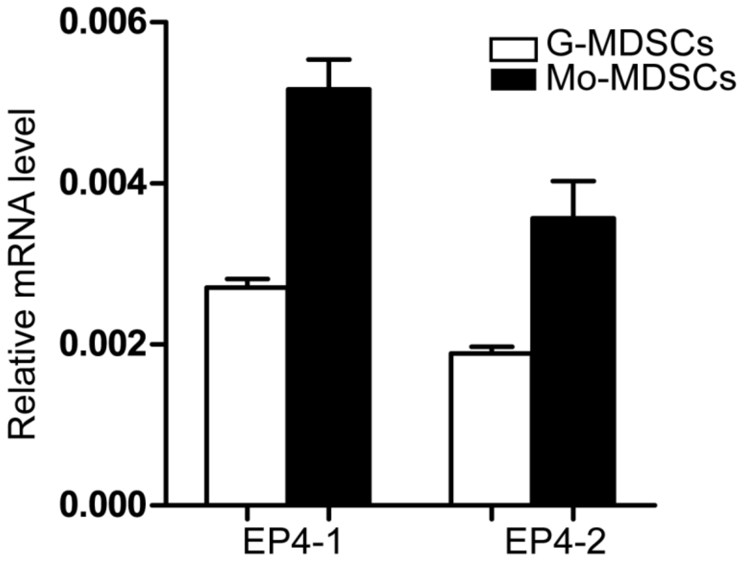

Supplementary Figure 1

EP4-1 and EP4-2 expression. G-MDSCs and Mo-MDSCs were sorted with MACS and *EP4-1* and *EP4-2* mRNA was measured with RT-PCR. Data are expressed as means ± SD relative to *GAPDH*.

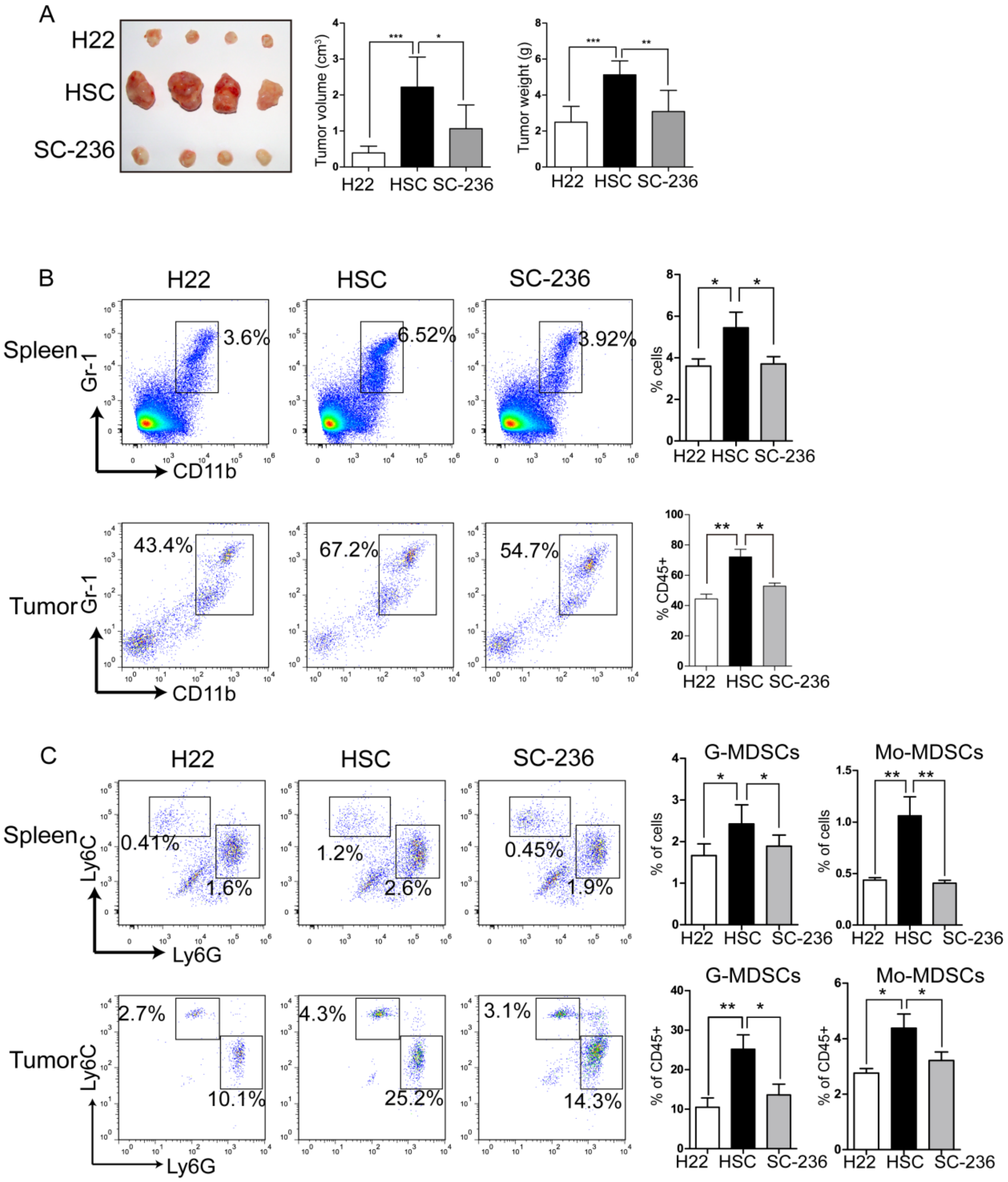

Supplementary Figure 2

HSCs promoted growth of HCC by induction of MDSCs via PGE_2 signaling. (A) Representative tumor samples, tumor volume and weight. (B) MDSCs in splenocytes and tumors were detected by flow cytometry; number is percent of the cell population represented by MDSCs (right

panels). Percent MDSCs was calculated using the formula: corrected MDSC percent = 100% × $CD11b^+$ $Gr\text{-}1^{+/low}$ percent × $CD45^+$ percent. (C) G-MDSCs and Mo-MDSCs were measured with flow cytometry. Number is percent of the cell population represented by G-MDSCs and Mo-MDSCs (right panels) and these were calculated as follows: corrected G-MDSC percent = 100% × $CD11b^+$ percent × $Ly6G^+Ly6C^{low}$ percent × $CD45^+$ percent. Corrected Mo-MDSC percent = 100% × $CD11b^+$ percent × $Ly6G^-Ly6C^{high}$ percent × $CD45^+$ percent. Data represent 3 independent experiments and are expressed as means ± SD; *$P < 0.05$, **$P < 0.01$, ***$P < 0.001$.

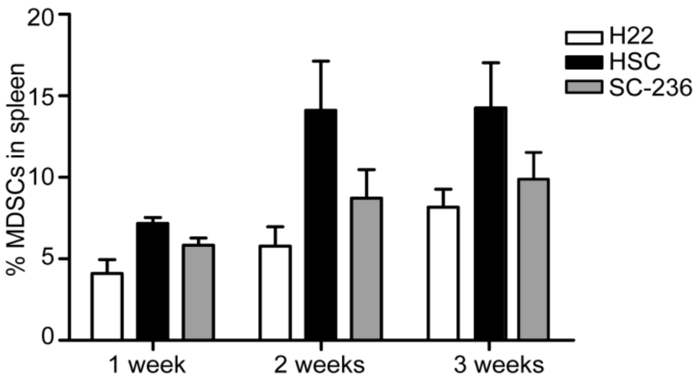

Supplementary Figure 3

Splenic MDSC populations in mice at 1, 2, and 3 weeks after tumor injection. Percent of MDSCs in spleen of tumor-bearing mice at indicated time. A subcutaneous mouse model was used to exclude increased MDSCs in the HSC group due to greater tumor burdens.

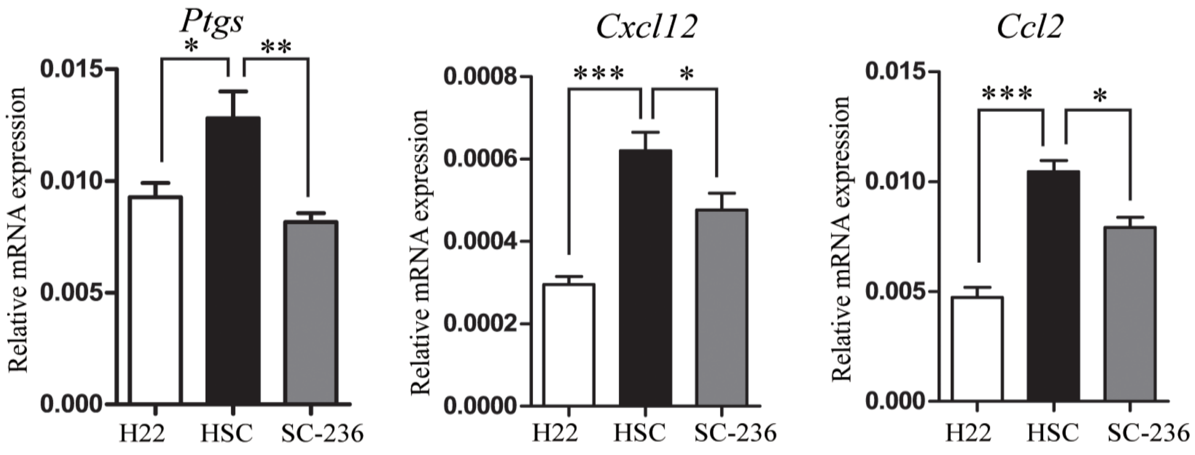

Supplementary Figure 4

Expression of cytokines and chemokines in the tumor microenvironment. The mRNA expression of *Ptgs2*, *Cxcl12*, and *Ccl2* in the tumor microenvironment was detected using RT-PCR.
